# 3′-sulfated Lewis^A/C^: An oncofetal epitope associated with metaplastic and oncogenic plasticity of the gastrointestinal foregut

**DOI:** 10.3389/fcell.2023.1089028

**Published:** 2023-02-14

**Authors:** Koushik K. Das, Jeffrey W. Brown

**Affiliations:** Division of Gastroenterology, Department of Medicine, Washington University in St. Louis, School of Medicine, St. Louis, MO, United States

**Keywords:** sulfation, glycosylation, barrett’s esophagus, gastric metaplasia, pancreatic intraepithelial neoplasia, pancreatic cancer, intraductal papillary mucinous neoplasm

## Abstract

Metaplasia, dysplasia, and cancer arise from normal epithelia *via* a plastic cellular transformation, typically in the setting of chronic inflammation. Such transformations are the focus of numerous studies that strive to identify the changes in RNA/Protein expression that drive such plasticity along with the contributions from the mesenchyme and immune cells. However, despite being widely utilized clinically as biomarkers for such transitions, the role of glycosylation epitopes is understudied in this context. Here, we explore 3′-Sulfo-Lewis A/C, a clinically validated biomarker for high-risk metaplasia and cancer throughout the gastrointestinal foregut: esophagus, stomach, and pancreas. We discuss the clinical correlation of sulfomucin expression with metaplastic and oncogenic transformation, as well as its synthesis, intracellular and extracellular receptors and suggest potential roles for 3′-Sulfo-Lewis A/C in contributing to and maintaining these malignant cellular transformations.

## Introduction

Cellular plasticity can be defined as a cell undergoing a phenotypic change resulting in a different histological appearance and RNA/Protein expression profile. This process occurs at homeostasis in tissues constitutively propagated by stem cells; however, more recently it has been shown to be a necessary aspect of tissue repair after injury. In the setting of prolonged/chronic injury and presumed mutational burden, plasticity allows tissue to transform into metaplasia as well as dysplasia, and cancer.

**FIGURE 1:**
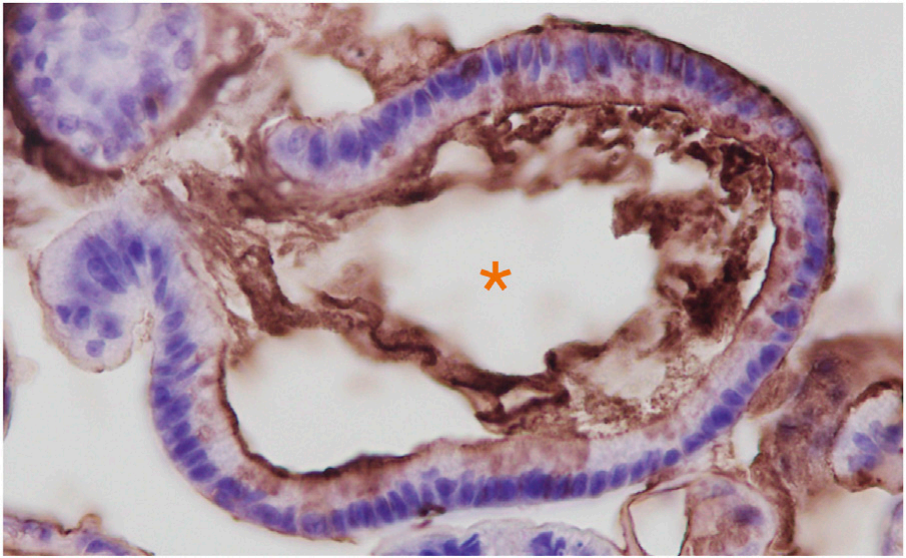
Anti-3′-Sulfo-Le^A/C^ (mAb Das-1) immunohistochemistry of Barrett’s esophageal organoids. Organoids were grown from endoscopic biopsies of individuals with Barrett’s esophagus as we have previously reported. (Jin et al., 2021). Immunohistochemistry of paraffin embedded organoids were stained as we have described in Brown et al., 2021a. Hematoxylin (blue) was used as a nuclear counterstain. Orange Asterix denotes the organoid lumen, which is bordered by the apical domain of the metaplastic cells through which the sulfomucins are being secreted.

Evidence for cellular plasticity of the GI foregut in injury and cancer largely derive from lineage tracing experiments using either Cre-reporters (Kopp et al., 2012; Han et al., 2019; Caldwell et al., 2022; Min et al., 2022) or pulse-chase experiments (Burclaff et al., 2020). Recently single-cell RNAseq (scRNAseq) trajectory/velocity analysis is also supportive of such transitions (Bockerstett et al., 2020a; Bockerstett et al., 2020b; Ma et al., 2022). Our knowledge of these metaplastic and oncologic tissue transitions derive primarily from *in vivo* murine studies due to several limitations of *in vitro* systems, namely: 1) the inability of cultured cancer cells or organoids from metaplastic tissue or cancer to reliably differentiate into homeostatic tissue with loss of cancer-associated transcription factors and glycosylation epitopes, (e.g. Figure 1) 2) the effects of growth conditions on organoids fueling constitutive proliferation reminiscent of dysplasia or cancer (Brown et al., 2022), 3) the lack of mesenchymal and immune interactions critical for metaplastic transition, (Petersen et al., 2014; Meyer et al., 2020; De Salvo et al., 2021), and 4) the lack of a deep mutational burden acquired over prolonged periods of inflammation (Baslan et al., 2022; Redston et al., 2022).

Clinically, these high-risk plastic cellular transformations are a hallmark of gastrointestinal pathology in practice. Indeed, while metaplastic transitions are prevalent in the setting of chronic inflammation in the form of Barrett’s esophagus, gastric intestinal metaplasia, and pancreatic intraepithelial neoplasia for example, they form the basis for dysplastic transformation into esophageal, gastric, and pancreatic adenocarcinomas, which have been recapitulated in murine models. Identification of patients at an early stage of metaplastic transition may allow for reversal (i.e. eradication of *H. pylori* infection in the stomach) or slowing (i.e. proton pump inhibitor therapy for Barrett’s esophagus). Similarly, by successfully identifying patients early in the metaplasia-to-dysplasia transition, minimally invasive endoscopic therapies such as endoscopic submucosal dissection of dysplastic gastric lesions or radiofrequency ablation of Barrett’s esophagus may be undertaken to potentially abrogate this process. The clinical challenge lies in identification of patients with fairly common metaplastic changes and subsequent risk stratification of patients with metaplasia who have undergone early stage dysplasia that requires intervention. For example, Barrett’s esophagus, prevalent in 1%–2% of the general population (Ronkainen et al., 2005) is readily identifiable with expensive, invasive, endoscopy; however, even once low-grade dysplasia has emerged the risk of cancer formation is <1% per year (Wani et al., 2011). While ever improving endoscopic and surgical approaches to dysplasia have emerged (Shaheen et al., 2022) and non-invasive means to assess the esophagus have been invented (Fitzgerald et al., 2020) the incidence of esophageal cancer has not significantly changed in the same period. Additionally unique challenges emerge for pancreatic intraepithelial neoplasia (PanIN) which is not readily detectable on radiographic modalities (Das and Early, 2017) or metaplasia of the stomach which may be endoscopically or histopathologically subtle to subtype.

Clearly, in the hopes of developing less invasive means of assaying for these high-risk plastic transitions, numerous groups are trying to identify biomarkers. Interestingly, nearly all commercially available serologic tests for metaplasia and cancer depend on assays for neoglycosylation epitopes and secreted glycoproteins (Table 1). However, there is a paucity of knowledge and understanding as to why cells begin to express and secrete these aberrant glycosylation epitopes and glycoproteins as they undergo plastic transitions to metaplasia and cancer. Further, there is a disconnect between the data produced from murine models of metaplasia, which primarily focuses on RNA/protein expression as well as the contributions of tumor microenvironment and clinically utilized assays for glycosylation epitopes and glycosylated proteins.

**TABLE 1:** List of serologic biomarkers used to diagnose and monitor cancer.

Biomarker	Type	Epitope	Cancer
AFP	Glycoprotein	Alpha Feto-Protein	Liver, Germ Cell Tumors
B-HCG	Glycoprotein	Human Chorionic Glonadotr	Seminoma, Choriocarcinoma, teratoma, germ cell tumors, Hydatidiform mole
B2M	Glycoprotein	Beta-2 microglobulin	Multiple Myeloma, Lymphoma
CA15-3	Glycoprotein	Muc1	Breast
CA125	Glycoprotein	Muc16	Ovarian
CA19-9	Glycosylation	3′-Sialyl-Lewis A	Pancreatic
CA27-28	Glycoprotein	Muc1	Breast
CA72-4	Glycoprotein	Large Mucin	Gastric, ovary, breast, colon, lung, pancreatic
CEA	Glycoprotein	CEACAM5	Colon, lung, breast, stomach, pancreas
PSA	Glycoprotein	Kallikrein-3	Prostate
S100	Protein	S-100	Melanoma

Here, we take a Top-Down approach: we start from a clinically validated biomarker, 3′-Sulfo-Le^A/C^, which has recently been recognized as the epitope of the antibody Das-1 (Brown et al., 2021a), for metaplasia and cancer throughout the GI foregut and review the literature in order to gain insight into how and why metaplasia and cancers might stereotypically express this antigen. With glycosylation epitopes, this approach is necessary because, as will be discussed, the cellular glycome cannot be readily predicted from cellular RNA/protein expression.

### Aberrant sulfation in metaplasia and cancer

Aberrantly sulfated glycosylation epitopes have long been recognized as a feature of high-risk metaplasia of the esophagus and stomach (Hakkinen et al., 1968; Bodger et al., 2003; Shah et al., 2020) as well as with dysplasia and cancer (Montero and Segura, 1980; Torrado et al., 1992; Fitzgerald et al., 2002). In the stomach, sulfation appears to be correlated with intestinal-type rather than diffuse-type of gastric cancer (Driessen et al., 1998).

Despite correlation with these plastic cellular transformations, sulfation remains poorly understood in part due to the paucity of commercial antibodies and lectins that are reactive towards it (McKitrick et al., 2021). Traditionally, histological identification of sulfated epitopes relies on high-iron diamine (HID) staining and specific Alcian blue stains at pH 1 (AB-1) (Reid et al., 1989; Padra and Linden, 2022). In addition to being agnostic to the moiety carrying the sulfate, these chemical stains are not compatible with more sophisticated imaging technologies like confocal or live cell imaging. ^35^S radiolabeling can be used to assay for sulfation; however, it has no spatial resolution within the tissue and again is agnostic to the moiety carrying the sulfate. Lastly, standard glycomic MS/MS analysis like collision-induced dissociation struggles to detect sulfate groups due to experimental loss with this technique (Kailemia et al., 2012).

**FIGURE 2:**
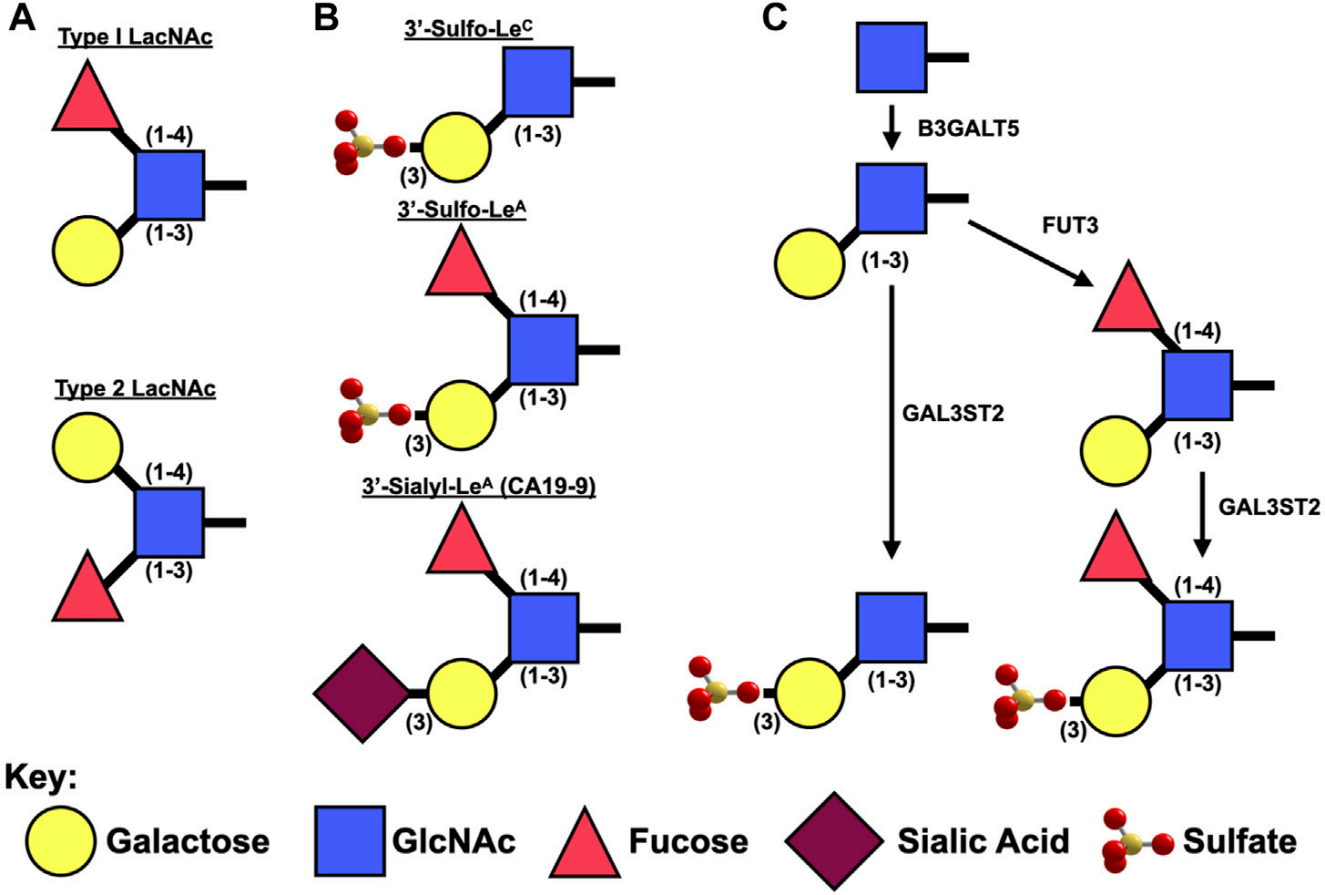
Synthesis of Type I Lewis epitopes. **(A).** Schematic representation of type 1 v. type II Lewis antigens. **(B).** Diagrams of sulfated and sialylated type I Lewis antigens. **(C).** Presumed synthetic pathway for 3′-Sulfated Le^A/C^.

Despite a lack of *commercially* available tools to detect sulfated glycans, historically, at least five groups have generated antibodies against 3′-Sulfated type I Lewis antigens (Table 2) and demonstrated that their reactivity phenocopies high-iron diamine staining in metaplasias and cancer of the gastrointestinal foregut (Das et al., 1987; Yamori et al., 1989; Rathman et al., 1990; Mitsuoka et al., 1998; Loveless et al., 2001). This suggests that at least a portion of the sulfated groups identified by HID/AB-1 staining represent 3′-Sulfo-Le^A/C^. Immunohistochemical surveys using these antibodies have demonstrated a consistent tissue distribution of 3′-Sulfo-Le^A/C^ in both normal fetal and adult tissue as well as during progression to metaplasia and cancer, cross-validating the specificity of these “homegrown” antibodies in tissue (Table 3). Three of these antibodies are specifically reactive towards 3′-Sulfo-Le^A/C^ [Das-1, F2, and 91.9H] and an additional two antibodies have affinity towards both 3′-Sulfo-Le^A/C^ and 3′-Sulfo-Le^X^ [SU59, MIN/3/60] (e.g. these latter reagents are unable to discriminate between Type I v Type II LacNAc; Figure 2 and Table 2).

**TABLE 2:** List of antibodies that are specifically reactive towards 3’-Sulfated galactose containing epitopes.

Antibody name	Species & Isotype	Known reactivity	Immunogen	First publication
Das-1 (7E12H12)	Mouse mAb IgMMouse mAb IgG1	3′-Sulfo-Le^A^3′-Sulfo-Le^C^	Extract from human colon	Das *et al*. *J Immunol* 1987; 139:77–84
F2	Mouse mAb IgM	3′-Sulfo-Le^A^3′-Sulfo-Le^C^	High Mr Mucins from human Saliva	Rathman *et al*. 1990 *J Biol Buccale* 1990; 18:19–27 (Journal no longer in existence)
91.9H	Mouse mAb IgG1	3′-Sulfo-Le^A^ in the setting of tetra-or pentasaccharide	Normal human colon	Yamori *et al*. *Cancer Res* 1989; 49:887–94
SU59	Mouse mAb IgM	3′-Sulfo-Le^A^3′-Sulfo-Le^X^	Unknown	Mitsuoka *et al*. *J Biol Chem* 1988; 273:11,225–33. Nisshin Shokuhin Co. Ltd., Otsu, Japan
MIN/3/60	Rat mAb IgM	3′-Sulfo-Le^A^ tetrasaccharide3′-Sulfo-Le^X^ tetrasaccharide	3′-SuLe^X^5 on *Salmonella* spp.	Loveless *et al*. *Hybridoma* 2001; 20: 223–9
O6	Chimera: Lamprey Variable Lymphocyte Receptor-Mouse IgG	3′-Sulfo-Le^X^3′-Sulfo-Gal-(1–4)-GlcNAc	Human Type O Erythrocytes	McKitrick *et al*. *Commun Biol* (2021) 4: 674
3′-Sulfo-Gal Containing Lipids				
M14-376*	Human mAb IgM	3′-Sulfo-Gal + Hydrophobic lipid Sulfated Glycosphingolipids	Human Lung Cancer	Miyake *et al*., *Cancer Res* 1992; 52:2292–7
Sulph I*	Mouse mAb IgG1	3′-Sulfo-Gal + Hydrophobic lipid	Glycolipid coated *Salmonella* spp.	Fredman *et al*., *Biochem J* 1988; 251: 17–22
OL-2	Rat mAb IgM	Sulfatide	Crude Rat Cerebellum	Colsch *et al*., *J Neuroimmunol* 2008; 193:52–8

**TABLE 3:** Tissue distribution of 3′-Sulfo-Le^A/C^ in fetal, adult, metaplastic, and cancerous tissue.

Fetal Distribution of 3’-Sulfo-Le^A^						
Tissue	Cell	Embryonic Origin	Organism	Antibody Used	Expression in Adult	References
Adrenal Gland	Cortex Cells	Ectodermal	Human	Das-1	No	Badve et al. (2000)
Appendix	Enterocyte	Endodermal/Hindgut	Human	Das-1	Yes	Badve et al. (2000)
Biliary	Bile ductal Cells, Gallbladder	Endodermal/Foregut	Human	Das-1	Yes	Badve et al. (2000); Das et al. (1992)
Colon	Enterocyte, Goblet Cell	Endodermal/Mid/Hindgut	Human	Das-1	Yes	Badve et al. (2000); Das et al. (1990), Das et al. (1992)
Esophagus	Epithelium	Endodermal/Foregut	Human	Das-1	No	Badve et al. (2000)
Kidney	Tubules = Collecting Duct	Mesodermal	Human	Das-1	No	Badve et al. (2000)
Liver	Hepatoblast, Ductal Plate Cells	Endodermal/Foregut	Human	Das-1	No	Badve et al. (2000)
Lung	Bronchiolar Epithelium > Alveoli	Endodermal/Foregut	Human	Das-1	No	Badve et al. (2000)
Pancreas	Islet > Acini	Endodermal/Foregut	Human	Das-1	No	Badve et al. (2000)
Small Intestine	Enterocytes	Endodermal/Midgut	Human	Das-1	No	Badve et al. (2000)
Stomach	Parietal Cells	Endodermal/Foregut	Human	Das-1	No	Badve et al. (2000)
Testis	Leydig Cells	Mesodermal	Human	Das-1	No	Badve et al. (2000)
Thymus	Hassal’s Corpuscles	Endodermal/Foregut	Human	Das-1	Yes	Badve et al. (2000)
Oropharynx	Oral = Pharyngeal	Endodermal/Foregut	Human	Das-1	No	Badve et al. (2000)
Skin	Keratinocyte	Ectodermal	Human	Das-1	Yes	Badve et al. (2000); Das et al. (1992)

Adult Distribution of 3’-Sulfo-Le^A^						
Tissue		Embryonic Origin	Organism	Antibody Used	References	
Extrahepatic Bile Duct		Endodermal Foregut	Human	Das-1	Das et al. (1990); Halstensen et al. (1993)	
Colon		Endodermal Mid/Hind Gut	Human, Tamarin, Rat, Mouse	Das-1, F2, 91.9H	* See Below	
Fallopian Tube		Mesodermal	Human	Das-1	Halstensen et al. (1993)	
Gallbladder		Endodermal Foregut	Human	Das-1	Das et al. (1990); Halstensen et al. (1993)	
Salivary Glands		Human	Human, Rat	F2	Veerman et al. (1997a)	
Skin		Ectodermal	Human	Das-1,F2	Das et al. (1990); Halstensen et al. (1993); Veerman et al. (1997a)	
Thymus (Hassal’s Corpuscles)		Endodermal Foregut	Human, Rat	F2	Veerman et al. (1997b)	

Re-expression of 3’-Sulfo-Le^A^ in Metaplasia and Cancer						
Tissue	Transformation		Organism	Antibody Used	References	
Bladder	Adenocarcinoma		Human	Das-1	Pantuck et al. (1997); Pantuck et al. (1998)	
Esophagus	Barrett’s Metaplasia		Human	Das-1, 91.9H	** See Below	
Esophagus	Adenocarcinoma		Human	Das-1	Das et al. (1994)	
Lung	Adenocarcinoma		Human	Das-1	Deshpande et al. (2002)	
Pancreas	Metaplasia (PanIN-3)		Human	Das-1	Das et al. (2021)	
Pancreas	High Grade Dysplasia and Adenocarcinoma		Human	Das-1	Onuma et al. (2001); Das et al. (2014); Das et al. (2019);	
Small Intestine	Adenocarcinoma (primarily with family history of AFP)		Human	Das-1	Onuma et al. (2001)	
Small Intestine	Adenocarcinoma		Human	Das-1	Onuma et al. (2001)	
Stomach	Metaplasia		Human	Das-1, F2, 91.9H	*** See Below	
Stomach	Adenocarcinoma		Human	Das-1, 91.9H	Ohe et al. (1994); Mirza et al. (2003); O'Connell et al. (2005)	
Urethral	Adenocarcinoma		Human	Das-1	Murphy et al. (1999)	

Decreased of 3’-Sulfo-Le^A^ in Metaplasia and Cancer						
Tissue	Transformation		Organism	Antibody Used	References	
Colon	Carcinoma		Human	91.9H	Matsushita et al. (1995)	
Intrahepatic Cholangiocarcinoma			Human	Das-1	Zimmerman et al. (2002)	

* (Das et al., 1987); (Das et al., 1990); (Biancone et al., 1991); (Das et al., 1992); (Halstensen et al., 1993); (Vermann et al., 1997a); (Yamori et al., 1989); (Irimura et al., 1991); (Yamachika et al., 1997); (Tsuiji et al., 1998a).

** (Bodger et al., 2003); (Das et al., 1994); (Glickman et al., 2001); (DeMeester et al., 2002); (Piazuelo et al., 2004); (Su et al., 2004); (Moriichi et al., 2009); (Hahn et al., 2009).

*** (Veerman et al., 1997a); (Bodger et al., 2003); (DeMeester et al., 2002); (Piazuelo et al., 2004); (Ohe et al., 1994); (Mirza et al., 2003); (Sun et al., 2006); (Watari et al., 2008); (Watari et al., 2012); (Feng et al., 2013).

Sulfatides (3-O galactosylceramide, e.g. SM4) are a class of sulfolipid that express a terminal 3′-Sulfo-Galactose, similar to 3′-Sulfated Lewis epitopes. Despite sharing the terminal 3′-Sulfo-Gal, antibodies recognizing sulfatides and 3′-Sulfo-Le^A/C^ have mutually exclusive tissue distributions. Anti-sulfatide antibodies are reactive towards kidney, pancreatic islets, and nervous tissue (Fredman et al., 1988; Miyake et al., 1992; Buschard et al., 1993a; Buschard et al., 1993b; Buschard et al., 1994; Colsch et al., 2008), which is distinct from the distribution for 3′-Sulfo-Le^A/C^ (Table 3). Thus although, the antibodies reactive to 3′-Sulfo-Le^A/C^ require a 3′-Sulfate on the terminal galactose they also require the adjacent glycans.

### Brief overview of protein glycosylation

Unlike protein expression, which usually correlates with RNA levels, protein glycosylation cannot be simply predicted from RNA or protein expression due to numerous competing factors. Following the initial glycosylation event in either the endoplasmic reticulum (for N-Linked glycans) or Golgi apparatus (for O-linked glycans), numerous enzymes compete with one another to add additional sugars (glycosyltransferases), remove sugars (glycosidases), and/or create branch points as the receptive moiety moves through the endoplasmic reticulum to the Golgi apparatus and beyond. These enzymes are not evenly distributed within each organelle, but instead are sorted to different regions and thus enzyme activity within the endoplasmic reticulum or cis-Golgi will affect the ability of more distally located enzymes in the trans-Golgi to act by changing the available glycosylation epitopes. In addition to differential expression of the proteins accepting these glycosylation epitopes, the speed, route of transport of the glycan acceptor, and availability of nucleotide-sugar donors will also differentially affect the end-product. Further, it is beginning to be appreciated that the activity of these glycosylation enzymes is also regulated (Li et al., 2022). Lastly, these glycans are modified not only by sulfotransferases (discussed here), but also by acetyl- and methyl transferases creating even greater diversity. Thus, even if the activity and donor specificity of each relevant glycosylation enzyme is known, it is essentially impossible to accurately predict a cellular glycome and this results in the production of heterogeneous products in most situations.

With all this complexity, it might be surprising that specific glycosylation epitopes are stereotypically expressed during specific plastic cellular transitions. However, the utility of such epitopes is clearly demonstrated by their clinical use as biomarkers to 1) diagnose cancer, 2) stage cancer, 3) monitor therapeutic response and/or 4) evaluate for recurrence (Table 1). While the synthesis, cellular effects, and tissue/organismal effects of 3′-Sulfo-Le^A/C^ is discussed here, much research is required to understand how and why such epitopes are stereotypically produced and secreted during these cellular transformations across many divergent organs.

### Synthesis and regulation of type 1 lewis epitopes (Le^C^ and Le^A^)

Le^A/C^ are type 1 Lewis antigens: they contain an N-Acetyl Glucosamine (GlcNAc) bound to a galactose *via* a β1-3 linkage (Figure 2). Lewis A differs from Lewis C in that it also has a fucose attached to GlcNAc *via* a one to four linkage. Type 1 Lewis antigens have been reported on proteins by both N- and O-Linked glycans as well as on glycosphingolipids.

In humans, there are five enzymes (β3GALT-1,-2,-4,-5,-6) that attach a galactose *via* a β3-link. Sequence-based phylogenetic analysis suggests that these enzymes segregate into three separate groups 1) β3GALT-1,-2, and,-5, 2) β3GALT-4, and 3) β3GALT-6, which parallel the preferred acceptor glycan (Togayachi et al., 2006) (Supplementary Image S1). β3GALT-4 is responsible for transferring galactose to glycosphingolipids (Daniotti et al., 1999) as well as glycoylphosphatidylinositol (GPI) (Wang et al., 2020). β3GALT-6 adds galactose to xylose in glycosylaminoglycans (Bai et al., 2001). β3GALT-1,-2, and -5 are capable of producing type I Lewis antigens on N-linked glycans; however, only β3GALT5 (Zhou et al., 1999) appears capable of adding this to O-linked glycans (Holgersson and Lofling, 2006). Since, the expression of O-Linked sulfated mucins have been best described in plastic cellular transformations of the GI foregut and β3GALT5 is generally believed to be responsible for the production of type I Lewis antigens in GI cancers (Isshiki et al., 1999; Salvini et al., 2001) we will focus on this enzyme. Further, overexpression β3GALT5 (in conjunction with FUT3) was able to generate Sialyl-Le^A^ (CA19-9) in a doxycycline inducible murine model that resulted in pancreatitis and augmented the development of tumors in the *Kras* ^*LSL-G12D*^ background (Engle et al., 2019).

The expression of β3GALT5 is complex, being promoted by CDX1 and CDX2 and potentially HNF1α/β that acts on a retroviral LTR promoter present only in old world monkeys and humans (absent in mice and new world monkeys); however, it is unclear whether this later transcriptional promoter is relevant (Isshiki et al., 2003). In addition to transcriptional factors, epigenetic methylation appears to play an important role in expression of β3GALT5 (Aronica et al., 2017).

Unlike β3GALT5, which specifically synthesizes type 1 Lewis antigens, the remaining two enzymes (FUT3 and GAL3ST2) that are necessary to synthesize 3′-Sulfo-Lewis A act on both type 1 [Galβ(1–3)GlcNAc] and type 2 [Galβ(1–4)GlcNAc] backbones (Figure 2). *FUT3* is unique among the numerous fucosyltransferases in that it is the only one capable of adding an α1,4-linked fucose to GlcNAc for the creation of Lewis A. Interestingly in mice, *Fut3* is a non-functional pseudogene due to multiple frameshift and non-sense mutations (Gersten et al., 1995) and as such it is believed that mice are only able synthesize Le^C^ and not Le^A^. Expression of *Fut3* is under the control of at least NFκB (Norden et al., 2017) and c-Myc (Sakuma et al., 2012).

### 3′-sulfation of galactose

**FIGURE 3:**
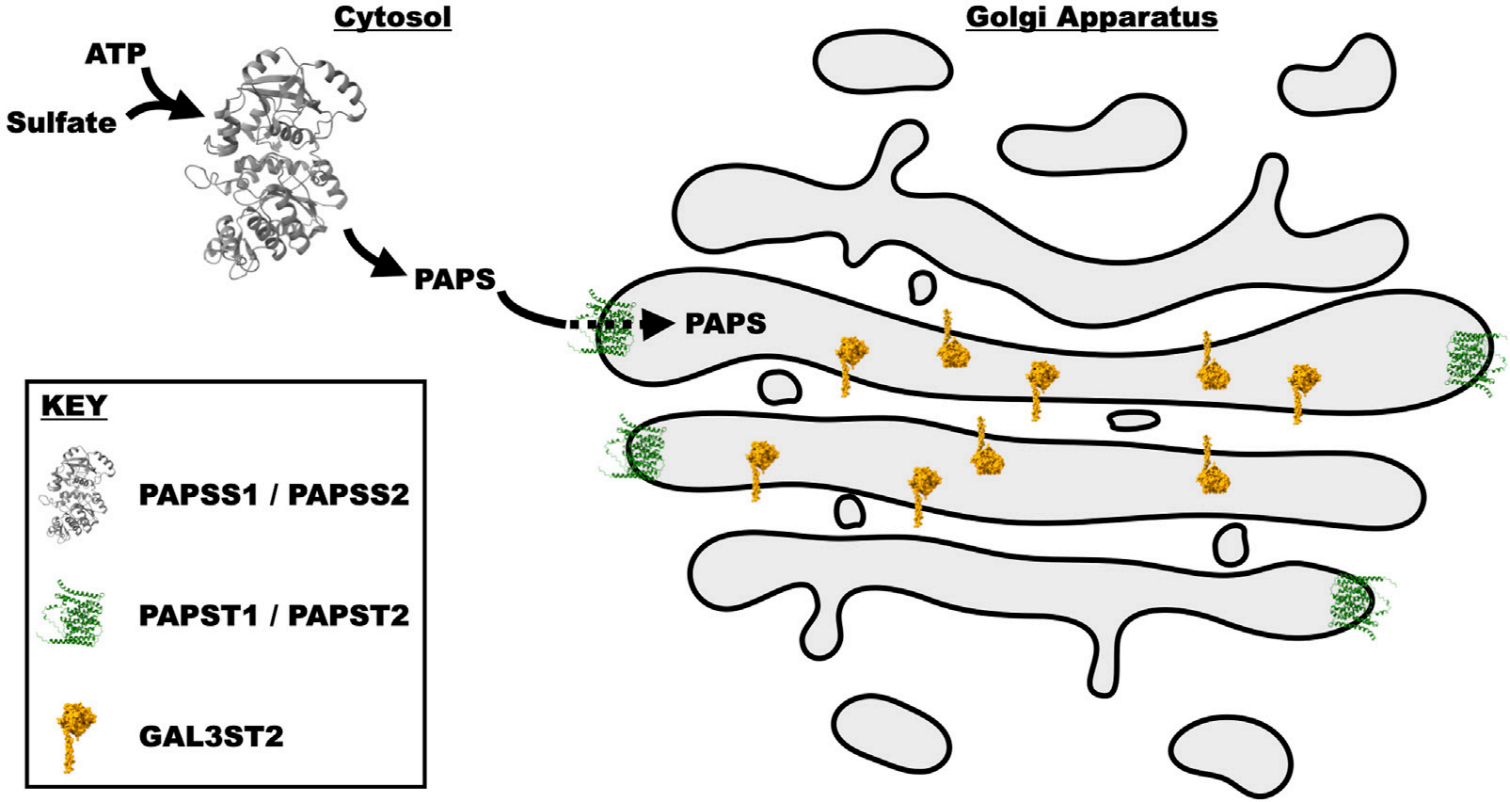
Schematic representation of the synthesis and transport of PAPS into the Golgi cisternae, where it is utilized by GAL3ST2 to sulfate Type I Lewis epitopes. PAPS is synthesized by the bifunctional enzymes PAPSS1 and PAPSS2. These paralogous proteins initially utilize the ATP sulfurylase domain to produce APS (Adenosine-5′-phosphosulfate). The APS intermediate is then acted upon by the APS kinase domain to generate PAPS. PAPS is subsequently transported from the cytosol into the Golgi lumen by two transporters PAPST1 and PAPST2. In the Golgi, PAPS is utilized by multiple different enzymes, one of which is GAL3ST2 that transfers the sulfate from PAPS to the 3′-site of galactose. These proteins were drawn in the medial Golgi with artistic license, as it is unclear where within the Golgi they reside. All structures were generated from the atomic coordinates utilizing the program chimera (Pettersen et al., 2004). The coordinates of PAPSS2 where obtained from PDB 7FHA (Zhang et al., 2022), while those for PAPSS1 and GAL3ST2 were predicted by the program AlphaFold (Jumper et al., 2021) and downloaded from the AlphaFold webserver (Varadi et al., 2022).

3′-Phosphoadenosine 5′-phosphosulfate (PAPS) is a universal sulfate donor for all sulfation reactions: those on glycans and those on aglycone moieties like tyrosine. This high-energy donor is generated from ATP *via* two, bifunctional homologous enzymes, PAPSS1 and PAPSS2 (Venkatachalam, 2003). PAPSS1 is reported to be located in the nucleus (Besset et al., 2000), while PAPSS2 is predominantly cytoplasmic (Schroder et al., 2012). The relative contribution of cellular PAPS from these enzymes is unclear and may vary by cell type. Since PAPS is produced in the nucleocytoplasm and sulfation of glycans occurs within vesicular compartments, there are two PAPS transporters (PAPST1 and PAPST2) on the Golgi membrane (Kamiyama et al., 2003; Kamiyama et al., 2006). Knockdown of PAPST1 and PAPST2 in DLD-1 cells (a cell line known to express 3′-Sulfo-Le^A/C^ (Hassan et al., 1995)) was shown to decrease sulfate incorporation not only in glycoproteins, but also chondroitin sulfate and heparan sulfate (Kamiyama et al., 2011) (Figure 3).

In both humans and mice, there are four distinct sulfotranferases capable of transferring a sulfate from PAPS to the 3′-site of a terminal galactose (*GAL3ST1* (Honke et al., 1997), *GAL3ST2* (Honke et al., 2001), *GAL3ST3* (El-Fasakhany et al., 2001; Suzuki et al., 2001), and *GAL3ST4* (Seko et al., 2001)). Like the galactosyltransferases, these enzymes have different tissue expression profiles and preferred glycan acceptors. It should also be noted that, in the 1990s when these genes were being cloned, the nomenclature was not standardized: *GAL3ST3* had been briefly called *GAL3ST2* (El-Fasakhany et al., 2001) and likewise *GAL3ST2* was called *GP3ST* (Honke et al., 2001; Ikeda et al., 2001), and *GAL3ST1* referred to as *CST* in several publications (cerebroside 3′-sulfotransferase). Each of these sulfotransferases have a unique acceptor specificity with GAL3ST2 being the only enzyme with strong activity toward Type I Lewis antigens (Honke et al., 2001; Suzuki et al., 2001; Seko et al., 2002). There has been a report of weak activity of GAL3ST3 toward Type 1 Lewis antigens; however, this might be due to cross-reactivity of the F2 antibody used (El-Fasakhany et al., 2001) as others were not able to detect activity when measuring transfer to pure substrates (Suzuki et al., 2001). GAL3ST1 is reportedly exclusively active toward ceramides (Honke et al., 1997), while GAL3ST4 has no activity against Galβ(1–3)GlcNAc (Seko et al., 2001). Thus GAL3ST2 appears to be the 3′-Sulfotransferase responsible for generating 3′-Sulfo-Lewis^A/C^. This enzyme is agnostic to the glycan carrier, being able to sulfate O-Linked, N-Linked, and glycolipids (Sun et al., 2022).

In mice, *Gal3st2* underwent a triplication into *Gal3st2*, *Gal3st2b*, and *Gal3st2c*, which exist in rapid succession over ∼110 kbp. Both the RNA and protein sequences are over 97% identical (Supplementary Image S2), which makes it virtually impossible to determine which of the three genes are translated or expressed by qPCR or other techniques. The transcriptional regulation of *GAL3ST2* is likewise unknown and some have argued that activity does not correlate with RNA/Protein levels and thus may be regulated by other means (Castro et al., 2012). Despite a relatively compact predicted structure by alpha fold (Supplementary Image S3), no high-resolution structural data exists for either of these enzymes or homologous proteins. Thus the molecular mechanism and critical residues by which they function remain to be determined as well as the possible regulation.

Immediately following the *GAL3ST2* gene in humans, mice, chicken, *etc.*, is *NEU4*, which removes a 2,3-linked sialic acid from Lewis A epitopes (Shiozaki et al., 2011). Databases like String (Szklarczyk et al., 2019) report that *GAL3ST2* and *NEU4* are coexpressed (Supplementary Image S4) potentially in *cis* on the same piece of mRNA (e.g. chimera) as this fusion transcripts has been reported in patent applications (https://patentimages.storage.googleapis.com/13/65/1e/27c585e9570bc4/US20160078168A1.pdf). as well as in cancer (Liu et al., 2021). Neu4 has been reported to be present in the endoplasmic reticulum and mitochondria (long isoform) (Yamaguchi et al., 2005; Bigi et al., 2010) as well as the lysosome (Seyrantepe et al., 2004). The lysosomal distribution is best supported by *in vitro* data as the enzymatic activity is negatively correlated with pH being most active at ∼3.2 (Monti et al., 2004), the pH of the lysosome. Mice lacking *Neu4* develop lysosomal storage pathology (Seyrantepe et al., 2008).

This location is interesting as autophagy has been demonstrated to be an essential aspect of plastic cellular transformation, specifically in the process of paligenosis (Willet et al., 2018; Brown et al., 2022). Potentially, sialylated proteins (e.g. those with 3′-Sialyl-Le^A^ e.g. CA19-9) could be shuttled to the lysosome where sialic acid is removed by the neuraminidase activity of Neu4, making the glycan amenable to sulfation. Despite being purely conjecture as the dependency of NEU4 on GAL3ST2 activity has never been reported, indirect evidence from tissue surveys of these plastic cellular transitions suggests a transition from sialylated to sulfated mucins as pathology progresses towards cancer. For example, the sole discriminating feature differentiating Type II intestinal metaplasia of the stomach from Type III is that the former expresses sialyated mucins and type III expresses sulfated mucins (Shah et al., 2020). Analogous to the stomach, acinar-to-ductal metaplasia and Pan-IN2 express 3′-Sialyl-Le^A^ (e.g. CA19-9) (Shi et al., 2014), which is in contrast to 3′-Sulfo-Le^A/C^ which is restricted to high-grade lesions (Pan-IN3 and PDAC) (Das et al., 2021). Thus, in multiple tissues the transition from sialylated to sulfated mucins (esp. involving Le^A/C^) correlates with the transition to higher risk metaplasia and cancer. Two potential explanations for the transition from sialylated-to-sulfated mucins are 1) increased expression of NEU4 and GAL3ST2 in *cis* or 2) altered vesicular transport routing mucins towards these enzymes. It remains to be determined whether either of these processes are required for the transition to sulfated mucins that correlate with greater incidence of having or developing foregut cancers.

### Fetal and adult tissue expression of 3′-Sulfo-Le^A/C^

While several groups have described the distribution of 3′-Sulfo-Le^A/C^ in mature adult tissue, a widespread survey of human fetal expression has only been described using the antibody Das-1 (formerly 7E_12_H_12_). In fetal tissue (analyzed at 7–22 weeks human gestational age), 3′-Sulfo-Le^A/C^ expression is widespread, but in many cases is restricted to a subset of cells in these organ systems (Table 3) (Das et al., 1987; Das et al., 1990; Das et al., 1992; Badve et al., 2000). In adult tissue at homeostasis, the 3′-Sulfo-Le^A/C^ epitope is not restricted to the germ layer and found in the colon (both enterocyte and goblet), extrahepatic biliary tract, MG1/MUC5B producing salivary glands, (Veerman et al., 2003), keratinocytes, and Hassall’s corpuscles of the thymus (Veerman et al., 1997b; Badve et al., 2000). In addition, low-grade patchy expression has been reported in the uterine cervix and submucosal glands of the lung (Veerman et al., 1997b).

### Re-expression of 3′-Sulfo-Le^A^ in metaplasia and cancer

Despite the absence of expression in the mature, adult esophagus, small intestine, pancreas, bladder, and lung, 3′-Sulfo-Le^A/C^ is re-expressed during the plastic cellular transformation into premalignant metaplasia and cancer (Tables 3, 4). As such, 3′-Sulfo-Le^A/C^ is a true oncofetal antigen in that expression is lost during maturation but reappears as cells transition into metaplasia and cancer. Re-expresssion of this antigen has been best studied in GI foregut: esophagus (Barrett’s esophagus and adenocarcinoma) (Das et al., 1994; Endo et al., 1998; Glickman et al., 2001; DeMeester et al., 2002; Bodger et al., 2003; Piazuelo et al., 2004; Su et al., 2004; Hahn et al., 2009; Moriichi et al., 2009), stomach (type III incomplete intestinal metaplasia and gastric adenocarcinoma) (Ohe et al., 1994; Veerman et al., 1997b; DeMeester et al., 2002; Bodger et al., 2003; Mirza et al., 2003; Piazuelo et al., 2004; O'Connell et al., 2005; Sun et al., 2006; Watari et al., 2008; Watari et al., 2012; Feng et al., 2013), and more recently by us in the exocrine pancreas [Pancreatic Intraepithelial Neoplasia (Pan-IN3), High-grade intraductal papillary mucinous neoplasm (IPMN), and pancreatic ductal adenocarcinoma (PDAC)] (Das et al., 2014; Das et al., 2019; Das et al., 2021).

**TABLE 4:** mAb Das-1 reactivity in gastrointestinal precancerous epithelial lesions and cancers derived therefrom.

	Reference	Normal Epithellum	Precancerous Condition	Sensitivity/Specificity	Cancer	Sensitivity/Specificity
Esophagus	Das et al. (1994); Piazuelo et al. (2003); Demeester et al. (2002); Watari et al. (2009)	Negative	Barrett’s Esophagus	95/100%	Esophageal Adenocarcinoma	100%100%
Stomach	Mirza et al. (2003); Piazuelo et al. (2003); Watari et al. (2012)	Negative	Gastric Intestinal Metaplasia incomplate type	35/100%*	Gasttic Adenocarcinoma	93%100%
Small intestine	Onuma et al. (2001)	Negative	Adenoma	50/100%	Small Bowel Adenocarcinoma	100%100%
Pancreas(PanIN)	Das et al. (2021)	Negative	PanIN	**	Pancreatic Adenocarcinoma	72%100%**
Pancreas(IPMN)	Das et al. (2014); Das et al. (2019)	Negative	IPMN	***	Pancreatic Adenocarcinoma	89%100%***

PanIN=Pancreatic intraepithelial Neoplasia; IPMN=Intraductal Papillary Mucinous Neoplasm

*Sensitivity & Specificity of Das -1 in non-gastric carcinoma associated gastric intestinal metaplasia(GIM) The low sensitivity is in part due to Das-1only being reactive to type lll incomplete intentinal Metaplasia and not Type ll incomplete intentinal Metaplasia.In GIM associated with Gastric Cancer sensitivity Specificity was 93% and 100% respectively

**Sensitivity & Specificity of Das-1 in segregating high-grade PanIN3/Adenocarcinoma from low-grade PanIN lesions(PanIN-1,PanIN-2)

***Sensitivity & Specificity of Das -1 in IPMN-associated cyst fluid by ELISA for segregating high-risk/invasive IPMN (invasive carcinoma, high-grade dysplasia of any epithelial subtype or intermediate grade dysplasia (IGD) of intestinal type) from low risk IPMN (gastric type IPMN with low grade dysplasla or IGD)

As previously discussed, expression of 3′-Sulfo-Le^A/C^ appears to be restricted to “higher-risk” histology relative to sialylated glycans. For example, sulfated mucins like 3′-Sulfo-Le^A/C^ are observed in type III intestinal metaplasia of the stomach as well as gastric adenocarcinoma (GC) (Bodger et al., 2003; Mirza et al., 2003), and by definition are not present in type I or II intestinal metaplasia of the stomach (Shah et al., 2020). Similarly, 3′-Sulfo-Le^A/C^ is restricted to Pan-IN3 and PDAC and not present in reversible lesions like acinar-to-ductal metaplasia or Pan-IN1 & Pan-IN2 (Das et al., 2021).

The corresponding sialic analog of 3′-Sulfo-Le^A^ is CA19-9 (3′-Sialyl-Le^A^) a widely used, commercially available biomarker. Although CA19-9 is a sensitive biomarker for the recurrence of pancreatic cancer after surgical resection or in response to chemotherapeutic treatment failure/non-response (Humphris et al., 2012), it can be non-specifically elevated in patients with and without pancreaticobiliary disease (Kim et al., 2020). In serum, elevated CA19-9 was observed in a minority of patients up to 2 years prior to the diagnosis of pancreatic ductal adenocarcinoma; however, this approached 60% sensitivity in 6 months preceding the diagnosis (Fahrmann et al., 2021). In patients with pancreatic cysts suspected of possible malignancy, serum CA19-9 has a sensitivity and specificity of 34.2% and 92.4%, respectively (Kim et al., 2015), which has been confirmed in larger studies and meta-analyses (Tanaka et al., 2019; Ciprani et al., 2020). Likewise, CA19-9 levels in cyst fluid perform poorly at differentiating benign IPMN from those harboring malignancy, with many studies demonstrating non-significant differences in the levels between these two cohorts (Nagashio et al., 2014; Kurita et al., 2019). In contrast, assaying for 3′-Sulfo-Le^A/C^ is 88% sensitive and 100% specific for identifying those cysts that harbor high-grade dysplasia and cancer (Das et al., 2014; Das et al., 2019). Thus, akin to the difference between Type II (Sialylated mucins) and Type III (Sulfated mucins) intestinal metaplasia of the stomach (Shah et al., 2020), in the pancreas sulfated mucins (3′-Sulfo-Le^A/C^) may be more sensitive and specific to high-grade dysplasia and cancer than the sialylated analogs (e.g. CA19-9).

**FIGURE 4:**
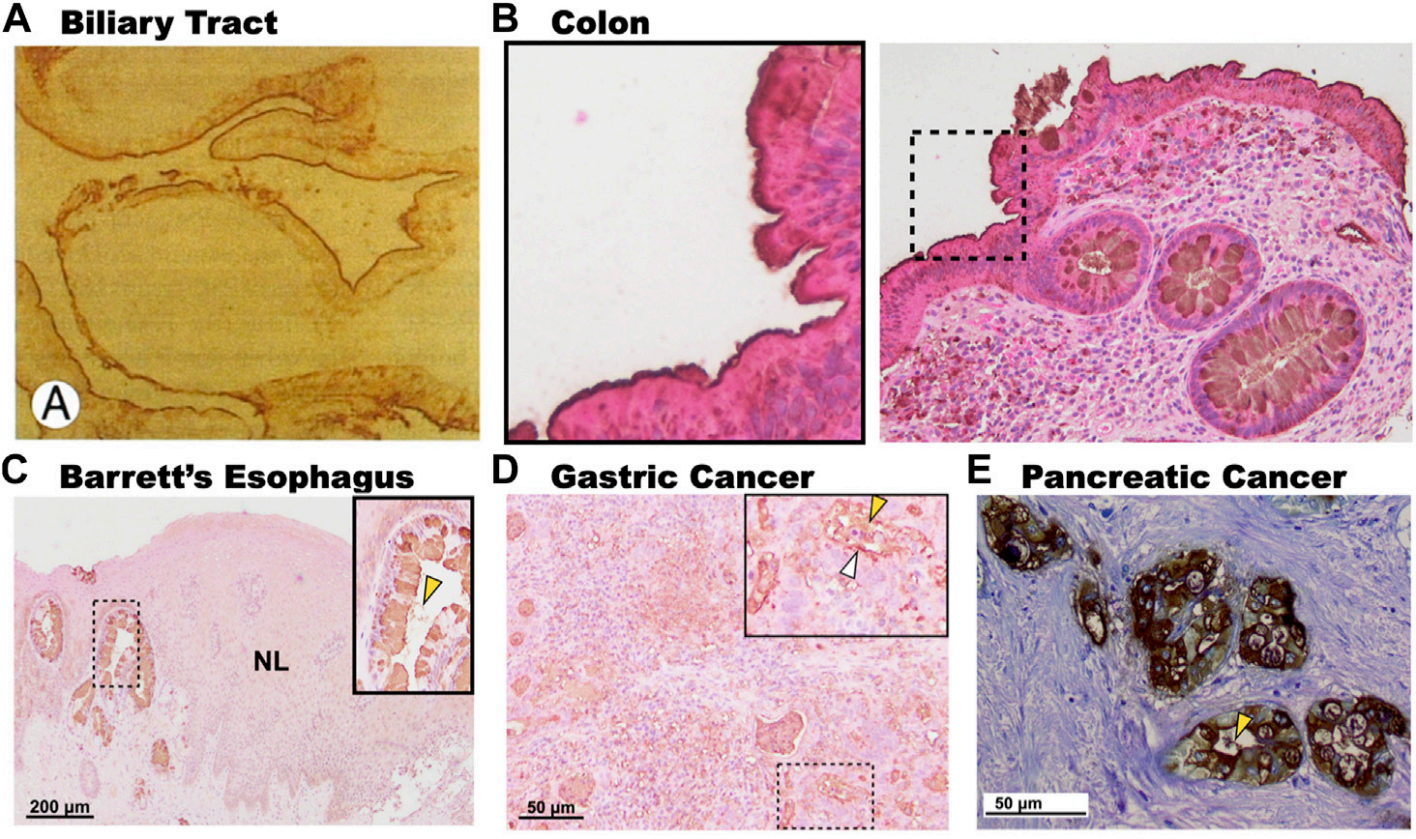
Subcellular expression patterns of 3′-Sulfo-Le^A/C^ in tissues at homeostasis and in metaplasia and cancer. **(A).** Immunohistochemistry of human gallbladder mucosa demonstrating expression of 3′-Sulfo-Le^A/C^ on the apical membrane of the biliary epithelium. **(B).** Expression of 3′-Sulfo-Le^A/C^ on the outer membrane of the colonic enterocytes (Inset), but intracellular in the goblet cells. In contrast, in metaplasia and cancer 3′-Sulfo-Le^A/C^ is absent from the apical membrane, being present intracellularly and is secreted as demonstrated in **(C).** Barrett’s Esophagus, **(D).** Gastric Cancer, and **(E).** Pancreatic cancer. Secretion denoted by yellow arrow arrowhead. (Panel A was reproduced with permission from Mandal et al., 1994; Panels C–E were reproduced from Brown et al., 2021a).

One notable difference between the expression of 3′-Sulfo-Le^A/C^ in normal columnar cells compared to cells that express 3′-Sulfo-Le^A/C^ only after plastic transition to metaplasia/dysplasia and cancer is the subcellular distribution. In normal columnar epithelial cells of the biliary tract and colon 3′-Sulfo-Le^A/C^ is primarily found on the apical extracellular membrane, presumably on the outer leaflet (Das et al., 1987; Das et al., 1992). (Figures 4A,B) In contrast, in metaplasia and cancer, the 3′-Sulfo-Le^A/C^ is expressed intracellularly and secreted being largely absent from extracellular membrane (Brown et al., 2021a) (Figure 1; Figures 4C–E). This secretion explains how we and others are able to detect 3′-Sulfo-Le^A^ in the serum and extracellular fluid upon plastic cellular transformation to high-grade dysplasia and cancer (Zheng et al., 2009; Das et al., 2014; Tanaka-Okamoto et al., 2017; Das et al., 2019). The reason for this different cellular distribution is unknown but almost certainly involves different vesicular trafficking pathways invoked during these plastic cellular transformations. Studying these divergent vesicular trafficking trajectories is an area of active investigation in our laboratory.

It is also interesting to note that expression of 3′-Sulfo-Le^A/C^ in human metaplasias often exhibits a variegated cell-by-cell expression where clonally-related neighboring cells may differentially express or not express the sulfomucins (Brown et al., 2021a; Das et al., 2021) (Figure 4C). Using synchronous mouse models, we believe that this occurs because of coordinated cellular process of expression and expulsion (Brown, 2021b; Brown, 2021c). Although secretion of sulfated mucins still occurs when cells progress from metaplasia to cancer, the variegated appearance is largely absent and all cells express similar quantities of the sulfated mucins (Brown et al., 2021a; Das et al., 2021) (Figure 4).

### Expression in mice and other mammalian Species

Precise epitope mapping suggests that at least two antibodies (F2 and Das-1) (Veerman et al., 1997b; Brown, 2021b) recognize the 3′-Sulfo-Galβ(1–3)GlcNAc (3′-Sulfo-Le^C^) chain and the fucose moiety of Le^A^ is not absolutely required; however, in some cases it may increase affinity. The independence of the α(1–4)fucose in Le^A^ becomes important when using mouse models to study the expression of this epitope because the sole alpha-4-fucosyltransferase (*Fut3*, Fut3) ortholog in mice is a non-functional pseudogene (Gersten et al., 1995). Thus, the consequences of 3′-Sulfation of Galβ1-3GlcNAc (Lewis C; Le^C^) in cells and at the tissue level can still be studied using the antibody Das-1 or the historically produced antibody F2 (Veerman et al., 1997b). In contrast, analysis of the epitope recognized by the historically generated 91.9H presumably requires both the fucose, as well as additional proximal sugars in the tetra- and pentasaccharide (Loveless et al., 1998). Using the *Kras* ^*LSL-G12D*^ *; TP53; Pdx1-cre* mouse, we have demonstrated that the expression of 3′-Sulfo-Le^C^ in mice (Brown et al., 2021a) parallels expression in humans (Das et al., 2021), being restricted to high-grade Pan-INs and pancreatic ductal adenocarcinoma. As such, genetic murine models appear to be a plausible tool to molecularly dissect how and why these sulfated mucins are expressed during the plastic transition from normal, homeostatic tissue into metaplasia, dysplasia, and cancer.

### Intracellular receptors for 3′-Sulfo-Le^A/C^

Galactose containing epitopes are generally recognized by a family of proteins called galectins (galactose binding lectins). At least three of the numerous galectins bind galactose containing epitopes that have a 3′-sulfate with greater or equal affinity: Galectin-3 (Ideo et al., 2002; Stowell et al., 2008; Xiao et al., 2018), Galectin-4 (Ideo et al., 2002; Ideo et al., 2005; Bum-Erdene et al., 2016; Xiao et al., 2018), and Galectin-8 (Ideo et al., 2003; Carlsson et al., 2007a; Carlsson et al., 2007b; Ideo et al., 2011; Xiao et al., 2018). It should be noted that in experiments the effect of 3′-Sulfation was tested compared to non-sulfated glycans (frequently with an adjacent GlcNAc), however, in none of these experiments was a fucose present in Lewis A. Thus, although these galactose binding motifs may prefer 3′-Sulfated galactose, it is unclear whether they bind 3′-Sulfo-Le^A^, or potentially just the afucosylalated 3′-Sulfo-Le^C^. The residues that are critical to recognizing the 3′-sulfate have begun to be investigated using the crystal structures of Galectins in complex with these sugars (Ideo et al., 2011; Bum-Erdene et al., 2015; 2016).

Two of the galectins that preferentially bind 3′-Sulfated galactose, Galectin-3 and -4, are among the 20 most upregulated proteins in gastric cancer relative to adjacent non-cancerous tissue (Zhang et al., 2021). The simultaneous expression of sulfomucins and their cognate galectins, suggests an intracellular function. In cell lines, when Galectin-3 and Galectin-8 recognize their glycans, they route the associated vesicular compartments to the lysosome for degradation (Thurston et al., 2012; Jia et al., 2020a; b). In addition to its role in stimulating autophagy, Galectin-8 has also been shown to modulate mTORC1 activity through the ragulator complex (Jia et al., 2018). Both autophagy and mTORC are essential components that are reciprocally regulated in paligenosis, a conserved process by which normal, homeostatic cells transform into metaplasia (Willet et al., 2018; Brown et al., 2022).

### Endogenous extracellular receptors for 3′-Sulfo-Le^A/C^

In the extracellular milieu, 3′-Sulfo-Le^A/C^ has been shown to be a potent ligand for selectins. E.g.: E-Selectin (Yuen et al., 1992; Yuen et al., 1994; Wang et al., 2022), L-Selectin (Green et al., 1992; Galustian et al., 1997; Galustian et al., 1999; Galustian et al., 2002), and P-selectin (Galustian et al., 2002; Appeldoorn et al., 2005). Selectins are a group of proteins expressed on numerous immune cells (E− & L-Selectins), as well as activated endothelial cells and platelets (P-selectins). These proteins assist in homing of cells to proper locations as an initial step in chemotaxis.

In addition to being potent ligands for selectins, 3′-Sulfo-Le^A^ has also been shown to be a preferred ligand for another receptor on macrophages: the cysteine-rich domain of the macrophages mannose receptor (Leteux et al., 2000), as well as on dendritic cells: dendritic cell immunoreceptor (DCIR) (Bloem et al., 2014). Thus, the expression and secretion of 3′-Sulfo-Le^A/C^ likely is involved in shaping the response to pathogen associated molecular patterns (PAMPs) and danger associated molecular patterns (DAMPs) through association and activation of these receptors.

In this context, it is interesting to speculate that tissues which express 3′-Sulfo-Le^A/C^ on their surface at homeostasis (e.g. biliary tract and colon; Figures 4A,B) might reduce expression upon oncogenic transformation to evade the immune system. While metaplastic, dysplastic tissues and cancer that lack the epitope on their surface (Figures 4C–E), might secrete these sulfomucins to potentially release decoys to evade the immune recognition or modulate the tumor microenvironment.

### Non-host, extracellular receptors for 3′-Sulfo-Le^A/C^

In the stomach, *H. pylori* is the predominant cause of metaplastic transformation and gastric cancer. Salivary Muc5B containing 3′-Sulfo-Le^A/C^ has been demonstrated to be a ligand for *H. pylori* (Veerman et al., 1997a). The interaction between these sulfated mucins and *H. pylori* has been demonstrated to be sensitive to pH such that binding is increased in the acidic milieu of the stomach. The *H. pylori* protein recognizing 3′-Sulfo-Le^A/C^ is Neutrophil Activating Protein (HP-NapA) (Teneberg et al., 1997; Namavar et al., 1998), which is a 17 kDa iron binding protein that oligomerizes into a dodecameric quaternary structure (Tonello et al., 1999). HP-NapA appears to bind the terminal 3′-Sulfo-Gal as affinity towards this truncated epitope is similar to that of 3′-Sulfo-Le^A^ (Veerman et al., 1997a; Namavar et al., 1998). Although levels may vary, HP-NapA is ubiquitously expressed among *H. pylori* stains (Evans et al., 1995) and appears to be located both in the cytosol (Namavar et al., 1998) and associated with the outer membrane vesicles after autolysis (Phadnis et al., 1996) analogous to virulence factors like Urease. Evidence for this mechanism comes from 1) HP-NapA has the exact same epitope specificity as was determined for *H. pylori* (3′-galactose and 3′-Sulfo-Le^A^) (Veerman et al., 1997a; Namavar et al., 1998) 2) vaccination of mice with HP-NapA protected the mice from subsequent challenge with *H. pylori* demonstrating that the protein was exposed to extracellular surface and an essential virulence factor (Satin et al., 2000). These data suggest that salivary sulfated Muc5B may associate with and potentially coat *H. pylori* and its associated OMV virulence factors limiting this pathogen’s ability to establish a niche in the stomach. The salivary Muc5B acting as a constitutively produced, prophylactic source of sulfated mucins, while induced expression and secretion during injury/metaplastic transformation is a secondary, reactionary source.

Distal to the GI foregut, secreted sulfated mucins have also been shown to be important in modulating commensal and pathogenic bacteria. Recently Xu and others have demonstrated that when synthesis of sulfated mucins are significantly reduced by intestinal deletion of *Papss2*, mice developed greater inflammation in murine models of colitis as well as a greater predilection to develop tumors (Xu et al., 2021). Others have also recently reported that bacteria have developed specific sulfatases towards 3′-Sulfo-Le^A^ (and other sulfated glycosylation epitopes) that are important in bacterial tropism within the colon (Luis et al., 2021).

### 3′-sulfation modulates cellular phenotype

Overexpression of GAL3ST2, the enzyme that adds the terminal 3′-Sulfate group to Galβ(1–3)GlcNAc, in 3T3L1 preadipocytes resulted in failure to differentiate as judged by accumulation of triglycerides and C/EBPβ expression (Guerra et al., 2015). These data suggest that 3′-Sulfo-Le^A/C^ may play a direct role in regulating cellular differentiation with expression favoring a dedifferentated phenotype, analogous to the transition from normal differentiated cells into metaplasia. Much more work needs to be done to elucidate the cellular phenotype elicited by the expression of sulfated mucins.

## Discussion and future directions

The stereotyped expression and secretion of 3′-Sulfated Le^A/C^ in high-risk metaplasia and cancer throughout the GI foregut (Table 4) has allowed several groups, including our own, to use it as both a histologic tool as well as biomarker for diagnosing these malignant cellular transformations. The utility of assaying for these sulfomucins is most important proximally as the human esophagus, stomach, and pancreas do not express this antigen under homeostatic conditions. This is in contrast to the colon and biliary tract, which express the epitope at homeostasis on their apical membrane, but reduce expression upon oncogenic transformation.

Despite the strong correlation of 3′-Sulfated Le^A/C^ with high-risk metaplasia and cancer in the gastrointestinal foregut, it is unknown why this epitope is stereotypically expressed. Although decreased expression of the enzymes necessary to synthesize 3′-Sulfo-Le^A/C^ correlates with the decreased expression in colon cancer, the contrapositive is not true as β3GALT5, FUT3, or GAL3ST2 do not appear to be significantly upregulated in all foregut cancers. As such, the expression of 3′-Sulfo-Le^A/C^ is potentially a consequence of altered vesicular trafficking or induction of autophagy, which are important aspects of these plastic cellular transitions. Alternatively, increased expression or differential expression of the mucins that accept these glycans as cells change their phenotype could also explain the different levels of these oncofetal epitopes. Even metabolic changes that occur in cancer could alter the availability of sulfate and/or activated sugars to assemble these epitopes.

Although it is known that the secreted sulfated mucins bind *H. pylori*, it is not known whether this increases the ability of *H. pylori* to penetrate deeper into the gland or is a protective mechanism e.g. coats the bacteria preventing them from adhering to the mucosa. The work in the colon might suggest that the later is likely the case as inhibition of sulfated mucins *via* intestinal specific knockout of *Papss2* results in greater injury and appears to augment carcinogenesis (Xu et al., 2021); however, these are different pathogens and colonic bacteria have even evolved sulfatases that function as colonization factors (Luis et al., 2021).

What also is not known is whether the expression of these sulfated epitopes are necessary for the plastic transition for metaplasia-to-dysplasia or cancer. A single study in cell lines suggested that the expression of sulfated mucins prevented differentiation, albeit in a non-epithelial lineage (Guerra et al., 2015). However, this in conjunction with the knowledge that the Galectins overexpressed in cancer (e.g. Galectin-3) and which bind this epitope induce autophagy an essential aspect of cellular plasticity, suggests that the expressions of these sulfated glycosylation epitopes might play a role in the cellular transformation and maintenance of the metaplastic, dysplastic, and oncogenic state. Further, the secreted sulfated mucins by way of immune receptors may play important roles in the tumor microenvironment. Overall, much work is needed to understand how and why specific glycosylation epitopes like 3′-Sulfo-Le^A/C^ are stereotypically expressed during transition into metaplasia, dysplasia, and cancer.

## Supplementary material

Supplementary image S1
**(A)** Phylogenetic tree of the five human B3GALTs computed using clustal omega software. **(B)** Uncurated multisequence alignment of the five human B3GALTs.

Supplementary image S2 Multisequence alignment of the murine triplicated Gal3st2 genes. Uncurated alignment computed with clustal omega (https://www.ebi.ac.uk/Tools/msa/clustalo/).

Supplementary image S3 Alpha fold prediction of human GAL3ST2. **(A,B)** Two views rotated 90 degrees about the Y-Axis. **(C)** The alpha fold confidence matrix computed for GAL3ST2 suggests that the transmembrane alpha helix and the globular domains were predicted with high confidence; however, the orientation (quaternary structure) of these two domains relative to one another is less certain.

Supplementary image S4 Reciprocal String-DB analysis of **(A)** GAL3ST2 and **(B)** NEU4 suggest that these genes are co-expressed.

## Abbreviations

HID, High-Iron Diamine; AB-1, Alcian Blue at pH 1; LeA, Lewis A; LeC, Lewis C; LeX, Lewis X, GIM, gastric intestinal metaplasia; PDAC, pancreatic adenocarcinoma; PAN-IN, pancreatic intraepithelial neoplasia; GC, gastric cancer.
