# Supplementary figures and images for: 3′-sulfated LewisA/C: An oncofetal epitope associated with metaplastic and oncogenic plasticity of the gastrointestinal foregut
Source: Front Cell Dev Biol. 2023 Feb 14;11:1089028. doi: 10.3389/fcell.2023.1089028 (PMC9971977; doi:10.3389/fcell.2023.1089028)

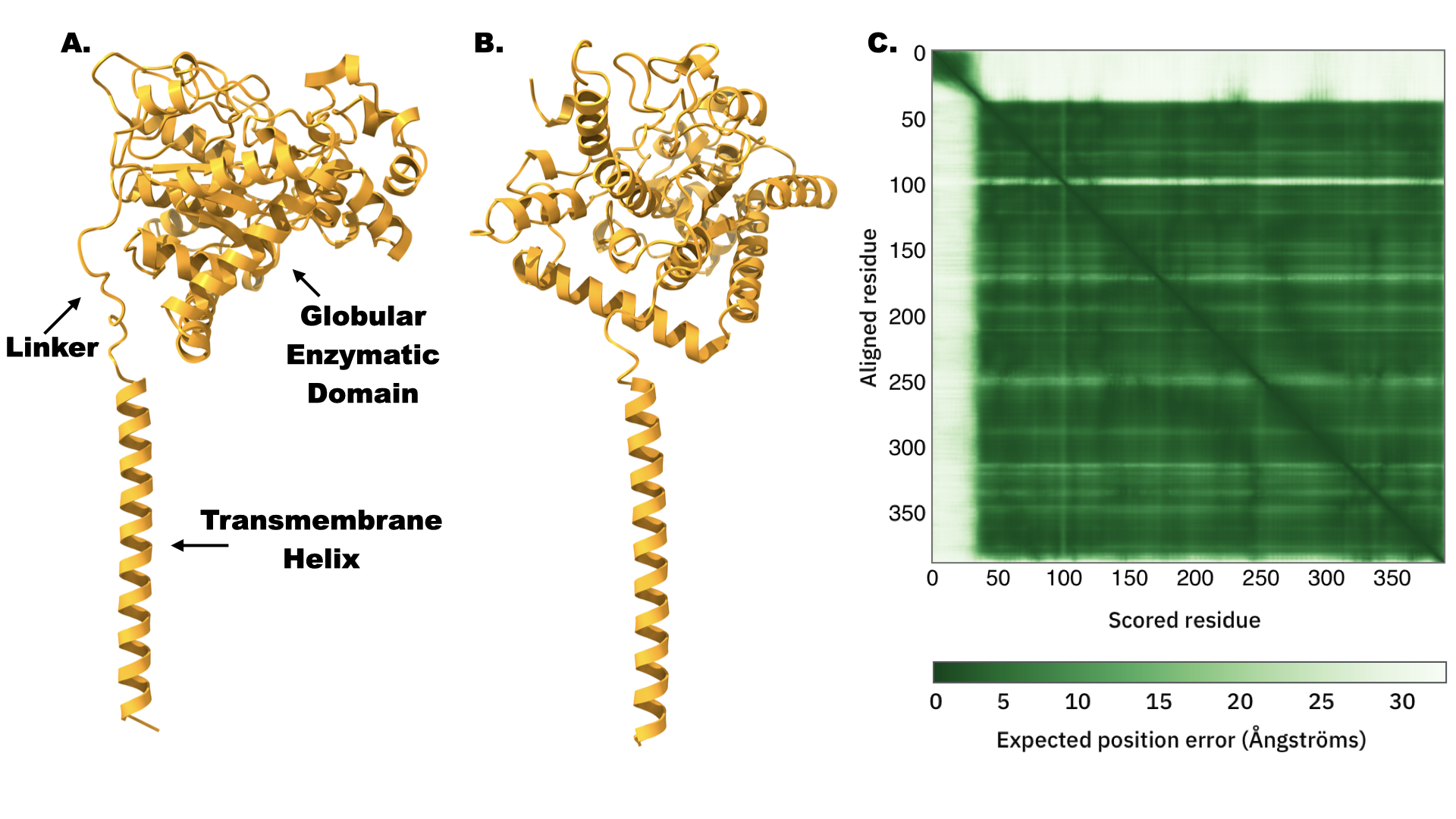

Supplement: Supplementary file 1 [file Image3.tiff]

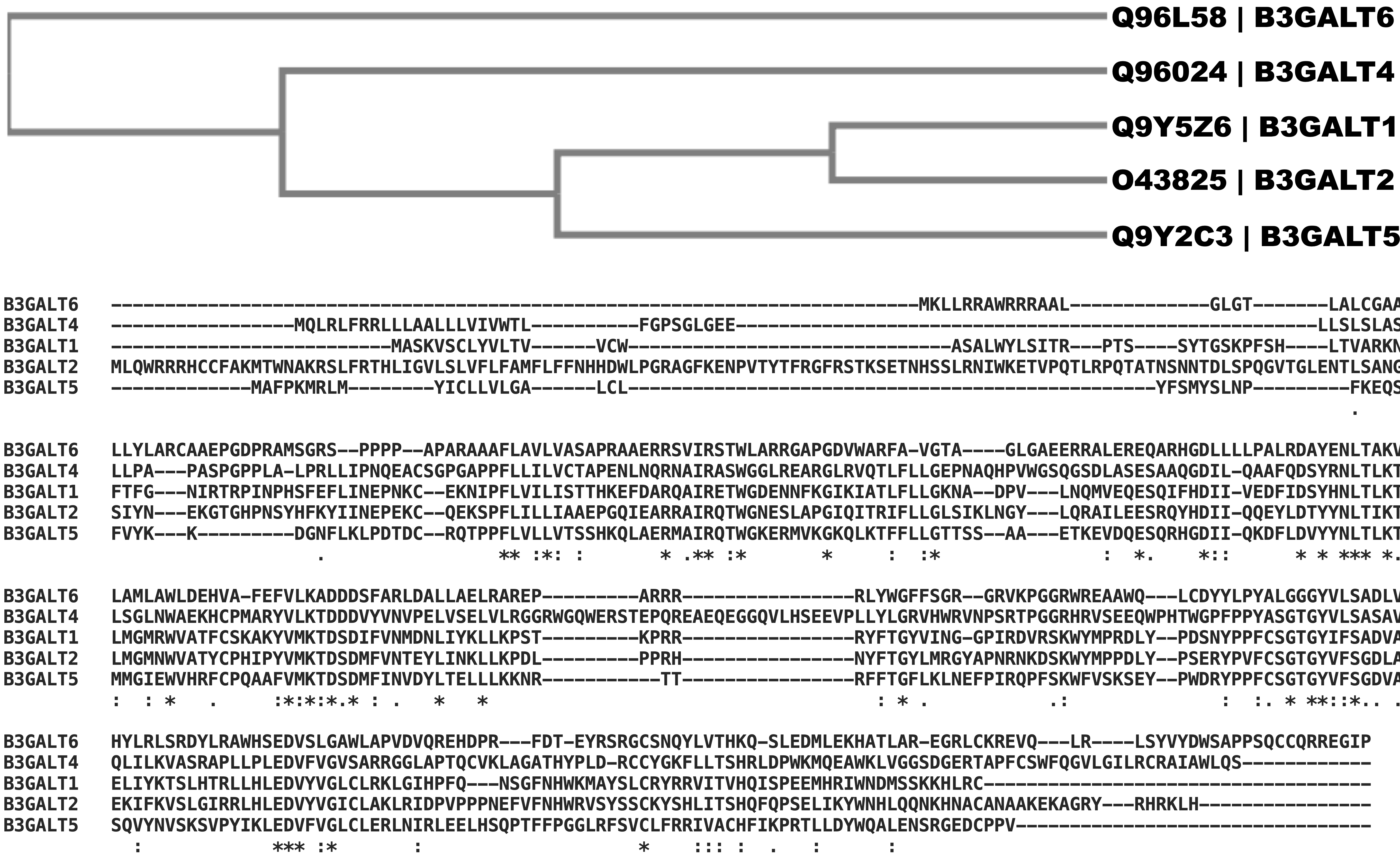

Supplement: Supplementary file 5 [file Image1.JPEG]

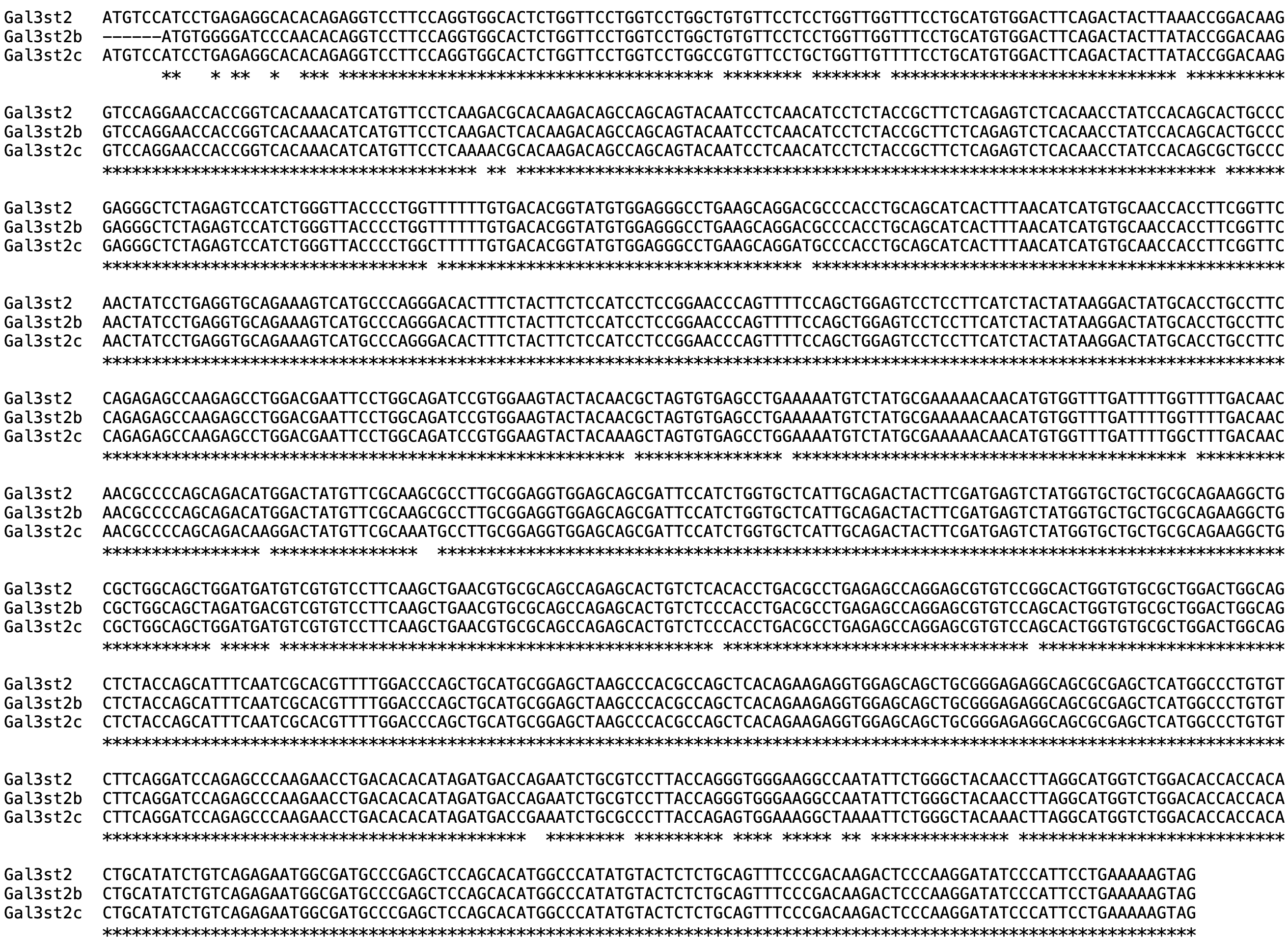

Supplement: Supplementary file 7 [file Image2.tiff]

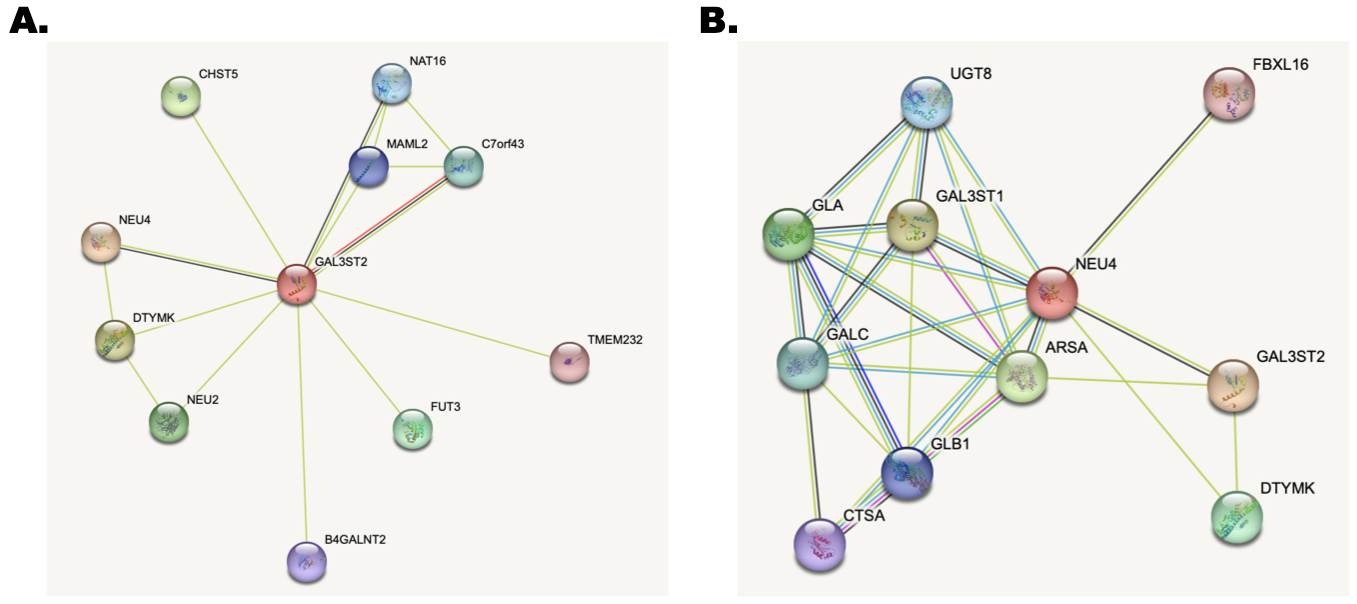

Supplement: Supplementary file 8 [file Image4.TIFF]
