# Supplementary material for: 3′-sulfated LewisA/C: An oncofetal epitope associated with metaplastic and oncogenic plasticity of the gastrointestinal foregut
Source: Front Cell Dev Biol. 2023 Feb 14;11:1089028. doi: 10.3389/fcell.2023.1089028 (PMC9971977; doi:10.3389/fcell.2023.1089028)
Supplement: Supplementary file 2 [file Table4.PDF]

	Reference	Normal Epithelium	Precancerous Condition	Sensitivity/ Specificity	Cancer	Sensitivity/ Specificity
**Esophagus**	Das KM et al Ann Intern Med 1994;Piazuelo et al Mod Pathol 2003; DeMeester et al Am J Gastro 2002; Watari et al Histopathology 2009	Negative	Barrett's Esophagus	95/100%	Esophageal Adenocarcinoma	100%/100%
**Stomach**	Mirza et al Gut 2003; Piazuelo et al Mod Pathol 2003; Watari et al CGH 2012.	Negative	Gastric Intestinal Metaplasia, incomplete type	35%/100%*	Gastric Adenocarcinoma	93%/100%
**Small Intestine**	Onuma et al Am J Gastro 2001	Negative	Adenoma	50%/100%	Small Bowel Adenocarcinoma	100%/100%
**Pancreas (PanIN)**	Das KK et al Human Pathology 2021	Negative	PanIN	**	Pancreatic Adenocarcinoma	72%/100%**
**Pancreas (IPMN)**	Das KK et al GUT 2014; Das KK et al Gastro 2019	Negative	IPMN	***	Pancreatic Adenocarcinoma	89%/100%***

PanIN = Pancreatic Intraepithelial Neoplasia; IPMN = Intraductal Papillary Mucinous Neoplasm

* Sensitivity &Specificity of Das-1 in non-gastric carcinoma associated gastric intestinal metaplasia (GIM). The low sensitivity is in part due to Das-1 only being reactive to Type III Incomplete Intestinal Metaplasia and not Type II Incomplete Intestinal Metaplasia. In GIM associated with Gastric Cancer, sensitivity/specificity was 93% and 100% respectively.

** Sensitivity & Specificity of Das-1 in segregating PanIN 3/Adenocarcinoma from low-grade PanIN lesions (PanIN-1, PanIN-2)

*** Sensitivity & Specificity of Das-1 in IPMN-associated cyst fluid by ELISA for segregating high risk/invasive IPMN (invasive carcinoma, high grade dysplasia of any epithelial type, or intermediate grade dysplasia (IGD) of intestinal type) from low-risk IPMN (gastric type IPMN with low grade dysplasia or IGD)
