# Supplementary material for: 3′-sulfated LewisA/C: An oncofetal epitope associated with metaplastic and oncogenic plasticity of the gastrointestinal foregut
Source: Front Cell Dev Biol. 2023 Feb 14;11:1089028. doi: 10.3389/fcell.2023.1089028 (PMC9971977; doi:10.3389/fcell.2023.1089028)
Supplement: Supplementary file 3 [file Table1.PDF]

Biomarker	Type	Epitope	Cancer
AFP	Glycoprotein	Alpha Feto-Protein	Liver, Germ Cell Tumors
B-HCG	Glycoprotein	Human Chorionic Glonadotr	Seminoma, Choriocarcinoma, teratoma, germ cell tumors, Hydatidiform mole
B2M	Glycoprotein	Beta-2 microglobulin	Multiple Myeloma, Lymphoma
CA15-3	Glycoprotein	Muc1	Breast
CA125	Glycoprotein	Muc16	Ovarian
CA19-9	Glycosylation	3'-Sialyl-Lewis A	Pancreatic
CA27-28	Glycoprotein	Muc1	Breast
CA72-4	Glycoprotein	Large Mucin	Gastric, ovary, breast, colon, lung, pancreatic
CEA	Glycoprotein	CEACAM5	Colon, lung, breast, stomach, pancreas
PSA	Glycoprotein	Kallikrein-3	Prostate
S100	Protein	S-100	Melanoma
