# Supplementary material for: 3′-sulfated LewisA/C: An oncofetal epitope associated with metaplastic and oncogenic plasticity of the gastrointestinal foregut
Source: Front Cell Dev Biol. 2023 Feb 14;11:1089028. doi: 10.3389/fcell.2023.1089028 (PMC9971977; doi:10.3389/fcell.2023.1089028)
Supplement: Supplementary file 4 [file Table2.PDF]

Antibody Name	Species & Isotype	Known Reactivity	Immunogen	First Publication
Das-1 ($7E_{12}H_{12}$)	Mouse mAb IgM<br>Mouse mAb IgG1	3'-Sulfo-Le^A<br>3'-Sulfo-Le^C	Extract from human colon	Das *et al*. *J Immunol* 1987; 139:77-84.
F2	Mouse mAb IgM	3'-Sulfo-Le^A<br>3'-Sulfo-Le^C	High M_r Mucins from human Saliva	Rathman *et al*. 1990 *J Biol Buccale* 1990;18:19-27 (Journal no longer in existence)
91.9H	Mouse mAb IgG1	3'-Sulfo-Le^A in the setting of tetra- or pentasaccharide	Normal human colon	Yamori *et al*. *Cancer Res* 1989; 49:887-94.
SU59	Mouse mAb IgM	3'-Sulfo-Le^A<br>3'-Sulfo-Le^X	Unknown	Mitsuoka *et al*. *J Biol Chem* 1988; 273:11225-33.<br>Nisshin Shokuhin Co. Ltd., Otsu, Japan
MIN/3/60	Rat mAb IgM	3'-Sulfo-Le^A tetrasaccharide<br>3'-Sulfo-Le^X tetrasaccharide	3'-SuLe^X5 on *Salmonella spp.*	Loveless *et al*. *Hybridoma* 2001; 20: 223-9.
O6	Chimera: Lamprey Variable Lymphocyte Receptor-Mouse IgG	3'-Sulfo-Le^X<br>3'-Sulfo-Gal-β(1-4)-GlcNAc	Human Type O Erythrocytes	McKitrick *et al*. *Commun Biol* (2021) 4: 674.
3'-Sulfo-Gal Containing Lipids				
M14-376*	Human mAb IgM	3'-Sulfo-Galβ + Hydrophobic lipid<br>Sulfated Glycosphingolipids	Human Lung Cancer	Miyake *et al*., *Cancer Res* 1992; 52:2292-7.
Sulph I*	Mouse mAb IgG1	3'-Sulfo-Galβ + Hydrophobic lipid	Glycolipid coated *Salmonella spp.*	Fredman *et al*., *Biochem J* 1988; 251: 17-22.
OL-2	Rat mAb IgM	Sulfatide	Crude Rat Cerebellum	Colsch *et al*., *J Neuroimmunol* 2008;193:52-8.
