# Supplementary material for: 3′-sulfated LewisA/C: An oncofetal epitope associated with metaplastic and oncogenic plasticity of the gastrointestinal foregut
Source: Front Cell Dev Biol. 2023 Feb 14;11:1089028. doi: 10.3389/fcell.2023.1089028 (PMC9971977; doi:10.3389/fcell.2023.1089028)
Supplement: Supplementary file 6 [file Table3.PDF]

Fetal Distribution of 3'-Sulfo-Le^A^

Tissue	Cell	Embryonic Origin	Organism	Antibody Used	Expression in Adult	References
Adrenal Gland	Cortex Cells	Ectodermal	Human	Das-1	No	Badve *et al.,* 2000
Appendix	Enterocyte	Endodermal/Hindgut	Human	Das-1	Yes	Badve *et al.,* 2000
Biliary	Bile ductal Cells, Gallbladder	Endodermal/Foregut	Human	Das-1	Yes	Badve *et al.,* 2000; Das *et al*., 1992
Colon	Enterocyte, Goblet Cell	Endodermal/Mid/Hindgut	Human	Das-1	Yes	Badve *et al.,* 2000; Das *et al*., 1990, Das *et al*., 1992
Esophagus	Epithelium	Endodermal/Foregut	Human	Das-1	No	Badve *et al.,* 2000
Kidney	Tubules = Collecting Duct	Mesodermal	Human	Das-1	No	Badve *et al.,* 2000
Liver	Hepatoblast, Ductal Plate Cells	Endodermal/Foregut	Human	Das-1	No	Badve *et al.,* 2000
Lung	Bronchiolar Epithelium > Alveoli	Endodermal/Foregut	Human	Das-1	No	Badve *et al.,* 2000
Pancreas	Islet > Acini	Endodermal/Foregut	Human	Das-1	No	Badve *et al.,* 2000
Small Intestine	Enterocytes	Endodermal/Midgut	Human	Das-1	No	Badve *et al.,* 2000
Stomach	Parietal Cells	Endodermal/Foregut	Human	Das-1	No	Badve *et al.,* 2000
Testis	Leydig Cells	Mesodermal	Human	Das-1	No	Badve *et al.,* 2000
Thymus	Hassal's Corpuscles	Endodermal/Foregut	Human	Das-1	Yes	Badve *et al.,* 2000
Oropharynx	Oral = Pharyngeal	Endodermal/Foregut	Human	Das-1	No	Badve *et al.,* 2000
Skin	Keratinocyte	Ectodermal	Human	Das-1	Yes	Badve *et al.,* 2000; Das *et al*., 1992

Adult Distribution of 3'-Sulfo-Le^A^

Tissue	Embryonic Origin	Organism	Antibody Used	References
Extrahepatic Bile Duct	Endodermal Foregut	Human	Das-1	Das *et al*., 1990; Halstensen *et al*., 1993
Colon	Endodermal Mid/Hind Gut	Human, Tamarin, Rat, Mouse	Das-1, F2, 91.9H	* See Below
Fallopian Tube	Mesodermal	Human	Das-1	Halstensen *et al*., 1993
Gallbladder	Endodermal Foregut	Human	Das-1	Das *et al*., 1990; Halstensen *et al*., 1993
Salivary Glands	Human	Human, Rat	F2	Veerman *et al*., 1997
Skin	Ectodermal	Human	Das-1,F2	Das *et al*., 1990; Halstensen *et al*., 1993; Veerman *et al*., 1997
Thymus (Hassal's Corpuscles)	Endodermal Foregut	Human, Rat	F2	Veerman *et al*., 1997

Re-expression of 3'-Sulfo-Le^A^ in Metaplasia and Cancer

Tissue	Transformation	Organism	Antibody Used	References
Bladder	Adenocarcinoma	Human	Das-1	Pantuck *et al*., 1997; Pantuck *et al*., 1998
Esophagus	Barrett's Metaplasia	Human	Das-1, 91.9H	** See Below
Esophagus	Adenocarcinoma	Human	Das-1	Das *et al*., 1994
Lung	Adenocarcinoma	Human	Das-1	Deshpande *et al*., 2002
Pancreas	Metaplasia (PanIN-3)	Human	Das-1	Das *et al*., 2021
Pancreas	High Grade Dysplasia and Adenocarcinoma	Human	Das-1	Onuma *et al*., 2001; Das *et al*., 2014; Das *et al*., 2019;
Small Intestine	Adenocarcinoma (primarily with family history of AFP)	Human	Das-1	Onuma *et al*., 2001
Small Intestine	Adenocarcinoma	Human	Das-1	Onuma *et al*., 2001
Stomach	Metaplasia	Human	Das-1, F2, 91.9H	*** See Below
Stomach	Adenocarcinoma	Human	Das-1, 91.9H	Ohe *et al*., 1994; Mirza *et al*., 2003; O'Connell *et al*., 2005
Urethral	Adenocarcinoma	Human	Das-1	Murphy *et al*., 1999

Decreased of 3'-Sulfo-Le^A^ in Metaplasia and Cancer

Tissue	Transformation	Organism	Antibody Used	References
Colon	Carcinoma	Human	91.9H	Matsushita *et al*., 1995
Intrahepatic Cholangiocarcinoma		Human	Das-1	Zimmerman *et al*., 2002

* Das *et al*., 1987; Das *et al*., 1990; Biancone *et al*.,1991; Das *et al*., 1992; Halstensen *et al*., 1993; Vermann *et al*., 1997; Yamori *et al*.,1989; Irimura *et al*., 1991; Yamachika *et al*.,1997; Tsuiji *et al*.,1998
** Bodger *et al*., 2003; Das *et al*., 1994; Glickman *et al*., 2001; DeMeester *et al*., 2002; Piazuelo *et al*., 2004; Su *et al*., 2004; Moriichi *et al*.,2009; Hahn *et al*., 2009
*** Veerman *et al*., 1997; Bodger *et al*., 2003; DeMeester *et al*., 2002; Piazuelo *et al*., 2004; Ohe *et al*., 1994; Mirza *et al*., 2003; Sun *et al*., 2006; Watari *et al*., 2008; Watari *et al*., 2012; Feng *et al*., 2013

Other
Regenerating Liver – look at reference
Ulcerative colitis?

References

1. Murphy DP, Pantuck AJ, Amenta PS, et al. Female urethral adenocarcinoma: immunohistochemical evidence of more than 1 tissue of origin. J Urol 1999;161:1881-4.
